# Frequency Characteristic of Resonant Micro Fluidic Chip for Oil Detection Based on Resistance Parameter

**DOI:** 10.3390/mi9070344

**Published:** 2018-07-09

**Authors:** Zilei Yu, Lin Zeng, Hongpeng Zhang, Guogang Yang, Wenqi Wang, Wanheng Zhang

**Affiliations:** Marine Engineering College, Dalian Maritime University, Dalian 110621, China; ray_yzl417@dlmu.edu.cn or yzl950417@126.com (Z.Y.); bobzl@dlmu.edu.cn (L.Z.); yanggg@dlmu.edu.cn (G.Y.); sarawang@dlmu.edu.cn (W.W.); zwanheng123@dlmu.edu.cn (W.Z.)

**Keywords:** resonant frequency, resistance parameter, micro fluidic, oil detection

## Abstract

Monitoring the working condition of hydraulic equipment is significance in industrial fields. The abnormal wear of the hydraulic system can be revealed by detecting the variety and size of micro metal debris in the hydraulic oil. We thus present the design and implementation of a micro detection system of hydraulic oil metal debris based on inductor capacitor (LC) resonant circuit in this paper. By changing the resonant frequency of the micro fluidic chip, we can detect the metal debris of hydraulic oil and analyze the sensitivity of the micro fluidic chip at different resonant frequencies. We then obtained the most suitable resonant frequency. The chip would generate a positive resistance pulse when the iron particles pass through the detection area and the sensitivity of the chip decreased with resonant frequency. The chip would generate a negative resistance pulse when the copper particles pass through the detection area and the sensitivity of the chip increased with resonant frequency. The experimental results show that the change of resonant frequency has a great effect on the copper particles and little on the iron particles. Thus, a relatively big resonant frequency can be selected for chip designing and testing. In practice, we can choose a relatively big resonant frequency in this micro fluidic chip designing. The resonant micro fluidic chip is capable of detecting 20–30 μm iron particles and 70–80 μm copper particles at 0.9 MHz resonant frequency.

## 1. Introduction

Hydraulic technology is widely applied in the country’s core industries, for example, aerospace, energy, and manufacturing industry due to its advantages of a large transmission force, flexible layout, and high efficiency. In the ship engineering field, hydraulic technology is always applied in major devices such as steering gears, propulsion systems, and lifting equipment. Hydraulic oil as the hydraulic system transmission medium is a kind of high cleanliness fluid, and more than 75% of hydraulic system failures are caused by hydraulic oil pollution [1]. Thus, predicting and diagnosing the contamination of hydraulic oil is the key to protecting the hydraulic system.

In hydraulic systems, hydraulic oil is inevitably contaminated due to internal generation and external intrusion, among which solid particle contamination is the main source of pollution. Some of these solid particle contaminants are external dust, and metal abrasive particles produced by internal mechanical wear. The latter is the most important cause of mechanical failure in hydraulic systems. The reference data [2,3,4] shows that the metal abrasive particles in the hydraulic oil have a constant concentration and the size of the abrasive particles is usually 10–20 µm when the hydraulic systems are under normal working conditions. On the contrary, when abnormal wear occurs in the hydraulic systems, the concentration of the metal abrasive particles in the hydraulic oil increases and the size of the particles becomes 50–100 µm. If the hydraulic system continues to work under this working condition, the concentration and size of the metal abrasive particles in the hydraulic oil will still increase until the hydraulic system fails. Thus, how to achieve the detection of particulate contaminants in hydraulic oil is an important content for predicting and diagnosing hydraulic oil contamination.

The main methods currently used for the detection of hydraulic oil include ferrous analysis, spectroscopic analysis, magnetic plug analysis, screen damping, particle counting, etc. [5]. With the exception of the particle counting method, several other methods can only estimate the pollution degree of the oil and require professional laboratory personnel to carry out analysis and measurement. Furthermore, these methods are difficult to achieve in oil on-line monitoring. The particle counting method is a common method for detecting fluid contamination, through the analysis of the amplitude and quantity of the pulses generated by the contaminants passing through the detection area, the size and number of the contaminants can be obtained and the accurate measurement of the oil contaminants can be truly achieved [6,7]. Further, the particle counting method can achieve on-line monitoring, so that it can better predict and diagnose the operation conditions of mechanical equipment and reduce time-consuming and labor-intensive shutdown inspections. Thus, this method has broad application prospects.

Currently, the combination of the micro fluidic electromagnetic technique and particle counting method can achieve some effects, mainly in the three following ways: (1) resistive pulse sensor (RPS), measuring the change of electrolyte resistance when a particle passes through micro channel [8,9,10], using multiple pores on a single chip as the detection area, detecting the resistance change when particles pass through the area; (2) capacitive counter sensor, measuring the difference in relativity permittivity between oil and metal particles [11,12], using opposite placement metal rods as the electrodes and detecting the changes when particles pass through the electrodes; and (3) inductive counter sensor, measuring the relative different permeability between ferrous particles, nonferrous metal particles, and non-metallic particles [13] using the inductance coil at high frequency condition to detect the particles. However, resistive pulse sensor detection is insensitive due to the fact that oil is non-conductive and non-isothermal. Capacitive counter sensor detection is interfered by a few water droplets in lubricant oil (the relativity permittivity of water and metal particles are both much larger than the relativity permittivity of oil), and it is also incapable of distinguishing the nonferrous metal particles and ferrous particles [14,15]. Du et al. demonstrated the feasibility and proposed a sensor using inductor capacitor (LC) resonant method based on the principle of such an inductive counter sensor and detected 32 μm iron particles and 75 μm copper particles with different excitation frequency by experiment [16,17]. Different from Du detection based on inductance parameter, in this paper, a resonant micro fluidic chip based on electromagnetic theory for detecting resistance parameter is designed and the frequency characteristic of a micro resistance sensor is explored. The theoretical calculations using MATLAB (MATLAB R2015a, The MathWorks, Natick, MA, USA) and experimental verification are performed that the change of resonant frequency has effect on detection. The resonant micro fluidic chip is capable of detecting 20–30 μm iron particles and 70–80 μm copper particles when the resonant frequency is 0.9 MHz, which improves the detection accuracy of ferromagnetic particles to some extent

## 2. Chip Design and Fabrication

**Figure 1:**
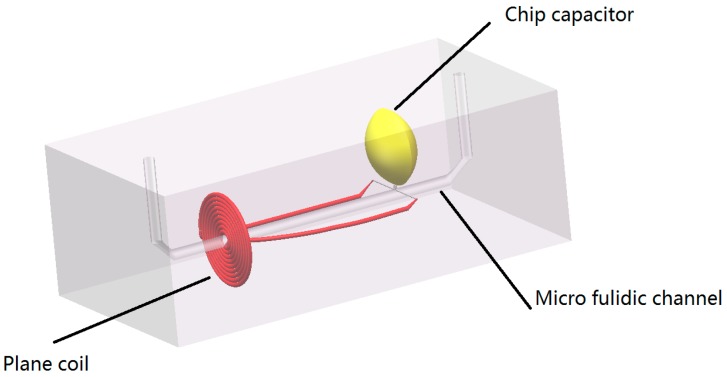
Micro fluidic resonant oil detection chip model figure.

The micro fluidic resonant oil detection chip is designed to detect the metal debris in oil based on electromagnetic theory as shown in Figure 1. The chip is composited of a plane coil, a chip capacitor, and a micro fluidic channel.

**Figure 2:**
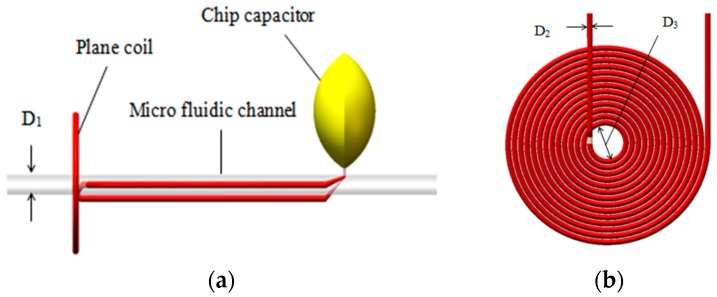
The design of micro fluidic resonant oil detection chip: (**a**) cross-section of the chip: *D*_1_ is the diameter of the micro-channel and (**b**) a sketch of a single-layer coil: *D*_2_ is the diameter of the coil wire core and *D*_3_ is the inner diameter of the single-layer coil.

The micro fluidic channel, which is close to the inner wall, goes across the inner hole of the coil, and the coil is connected with the capacitor in parallel. The diameter of micro-channel *D*_1_ is 300 μm (see Figure 2a). The diameter of the coil wire core *D*_2_ is 70 μm. There is a coat of insulating paint with a thickness of 10 μm around the coil wire core and the diameter of the coil inner hole *D*_3_ is 900 μm (see Figure 2b).

To fabricate the detection chip, a model of a coil connected with capacitors and a straight micro-channel was made first. The single-layer coil was made of enamel copper wire and wound by a winding machine (SRDZ23-1B, Zhongshan Shili Wire Winder Equipment Co., Ltd., Zhongshan, Guangdong, China); the number of turns is varied according to different experiments. The straight micro-channel model was made of an iron rod with a diameter of 300 μm and a length of 7 cm. It was put into the coil inner hole close to the inner wall (see Figure 2a). After that, both the micro-channel model, the capacitor, and the coil were fixed to a glass substrate using glue. The micro-channel model, the capacitor, the coil, and glass substrate formed a chip mold, upon which the liquid polydimethylsiloxane (PDMS) was poured. The chip mold was then placed in a thermostat with a temperature of 80 °C for 1 h. Finally, after the liquid PDMS was solidified, the straight micro-channel model was removed using pliers and the resonant oil detection chip fabrication was completed. The diameter of the micro-channel model equals to the diameter of the straight micro-channel.

## 3. Theoretical Analysis

The micro fluidic resonant oil detection chip presented in this paper is a resistance sensor, the change of the inductor coil causes the impedance of the entire circuit to change, and the chip detects the real part of the impedance change.

**Figure 3:**
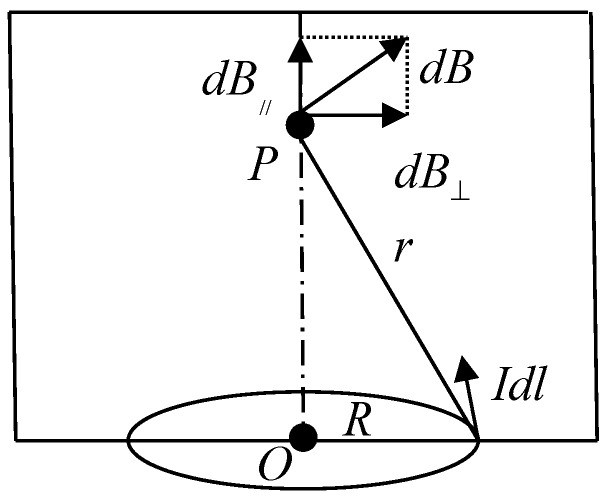
Magnetic field on the circular current-carrying conductor.

When an excitation voltage is applied to the coil, an alternating current is formed in the coil. Such a coil can be simplified as a circular current-carrying conductor and the magnetic field is shown in Figure 3. It is easy to know that the transverse components of the magnetic field generated by the coils cancel each other out and only have a magnetic field with an axial component. According to the Biot-Savart Law, the center of the coil has the highest magnetic induction. When metal particles pass through this position, the particles are magnetized and the coils generate increased magnetic flux; at the same time, particles will also generate eddy current effects at this position which will reduce the magnetic induction of the original magnetic field. For ferromagnetic particles, their relative magnetic permeability is much larger than 1, so when they pass through the detection area, the magnetization field inside them is much larger than the weakened magnetic field generated by the inner eddy current, and the apparent inductance of the coil increase. For non-ferromagnetic metal particles, the relative magnetic permeability is about equal to 1, so there is no magnetization effect. When the particles pass through the detection area, the magnetization field inside the particle is smaller than the weakened magnetic field generated by the inner eddy current, and the apparent inductance value of the coil reduce.

### 3.1. Particle Differentiate Detection

**Figure 4:**
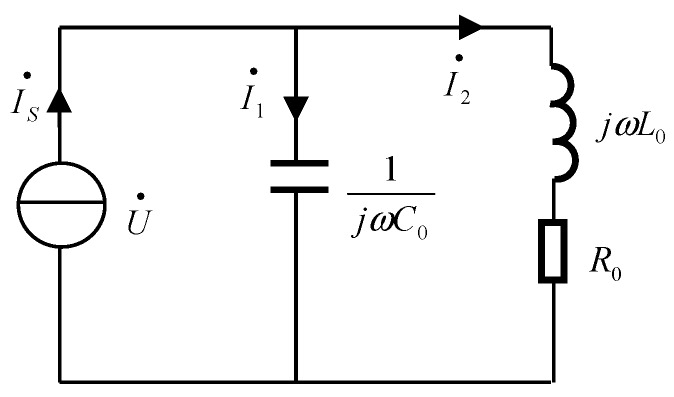
The equivalent circuit of micro fluidic resonant oil detection chip.

The equivalent circuit diagram of the micro fluidic oil detection chip described herein is shown in Figure 4. Among the figure, the inductor coil is equivalent to a pair of series inductance *L*_0_ and resistor *R*_0_, the entire circuit is composed of a series branch and a capacitor *C*_0_ in parallel. The resistance, inductance, and capacitance are expressed by a complex representation, the impedance of the entire circuit is *Z*_0_. According to the character of the parallel circuit, the branch connected in series with a resistor and an inductor corresponds to a single inductor coil with excitation voltage. Under a constant excitation, a time-harmonic magnetic field is generated and the magnetic induction intensity is constant. When the particles pass through the center of the coil, the particles are magnetized and generate an eddy current effect, resulting in a change in the apparent inductance value of the coil [18,19].

When the resonant angular frequency exists in a parallel resonant circuit, it is satisfied by $1-\frac{C_{0}R_{0}}{L_{0}}>0$ and it can be expressed as:

(1) $$\omega_{0}=\frac{1}{\sqrt{L_{0}C_{0}}}\sqrt{1-\frac{C_{0}R_{0}}{L_{0}}}$$

When the excitation frequency is the resonant frequency, the entire circuit presents a purely resistive state. When no particles pass through the detection area, the initial equation of the equivalent circuit can be expressed as:

(2) $$Z_{0}=\frac{R_{0}+j\omega_{0}L_{0}}{(1-\omega_{0}{}^{2}L_{0}C_{0})+j\omega_{0}R_{0}C_{0}}=\frac{R_{0}+j\omega_{0}(L_{0}-\omega_{0}{}^{2}L_{0}{}^{2}C_{0}-\omega_{0}R_{0}{}^{2}C_{0})}{{\left(1-\omega_{0}{}^{2}L_{0}C_{0}\right)}^{2}+\omega_{0}{}^{2}R_{0}{}^{2}C_{0}{}^{2}}$$

The real part of the impedance can be expressed as:

(3) Re(Z0)=R0(1−ω02L0C0)2+ω02R02C02

When there are metal particles passing through the detection area, the apparent inductance value of the coil changes as described above, so that the changed value produced is the inductance ∆*L*, and the impedance change value of the entire circuit is ∆*Z*. At this time, the real part of the changes can be expressed as:

(4) Re(Z0+ΔZ)=R0X12+X22

Among them:

(5) $$\left\{ \begin{array}{l} X_{1}={\left[1-\omega_{0}{}^{2}(L_{0}+\Delta L)C_{0}\right]}^{2}\\ X_{2}=\omega_{0}{}^{2}R_{0}{}^{2}C_{0}{}^{2} \end{array}\right.$$

Because only the inductance value has changed, only *X*_1_ has changed in Formula (5). As can be seen, the resonance angular frequency from the previous Formula (1), *X*_1_ can be expressed as:

(6) $$X_{1}={\left[1-\frac{1}{L_{0}}(1-\frac{C_{0}R_{0}{}^{2}}{L_{0}})(L_{0}+\Delta L)\right]}^{2}$$

It can be seen from the beginning that when ferromagnetic particles pass through the detection area, the apparent inductance of the coil increases, that is, ∆*L* > 0; and when the non-ferromagnetic metal particles pass through the detection area, the apparent inductance of the coil decreases, that is, ∆*L* < 0. The Formulas (3) and (6) show that when the ferromagnetic particles pass, *X*_1_ decreases and the real part of the circuit impedance increases. When non-ferromagnetic metal particles pass, *X*_1_ increases and the real part of the circuit impedance decreases. Therefore, ferromagnetic particles and non-ferromagnetic particles can be distinguished by detecting the real part of the entire circuit impedance.

### 3.2. Effect of Excitation Frequency on Detection

For a single coil micro fluidic chip, the frequency characteristics have been studied. Through the research, it has found that when the excitation frequency is lower than 2 MHz, the frequency change has little effect on the chip detection capability [20]. The inductor coil used in this paper has an inductance of approximately 10 μH and a resistance of approximately 0.8 Ω. In the previous study, our research team has found that a single coil chip was used for detection the 80–90 μm iron particles, the apparent inductance value generated by the inductor coil was changed approximately 4 × 10^−5^ μH. And for the 150–160 μm copper particles, the apparent inductance value generated by the inductor coil was changed approximately −3 × 10^−5^ μH. In this paper, the resonant frequency of the detection chip is changed by changing the value of the chip capacitor in parallel, and the frequency of the excitation voltage is set to the resonant frequency. From Formulas (3)–(5), the real part impedance signal amplitude can be expressed as:

(7) $$\Delta Z=\frac{R_{0}}{{\left[1-\omega^{2}(L_{0}+\Delta L)C\right]}^{2}+\omega^{2}R_{0}{}^{2}C^{2}}-\frac{R_{0}}{{\left(1-\omega^{2}L_{0}C\right)}^{2}+\omega^{2}R_{0}{}^{2}C^{2}}$$

Among them, the capacitor and the excitation angular frequency are variables, the resistance and the inductance of the inductor coil are constants. And the relationship between resonant frequency and resonant angular frequency is:

(8) $$f=\frac{\omega }{2\pi }$$

**Figure 5:**
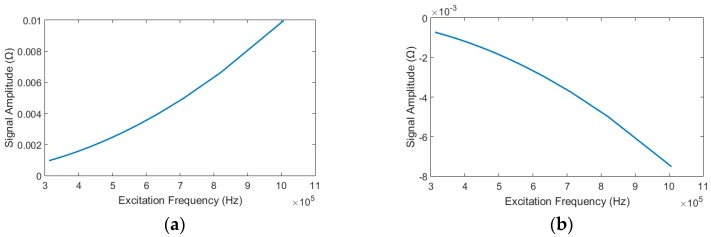
The relationship between excitation frequency and impedance signal amplitude value: (**a**) impedance signal value produced by ferromagnetic particle and (**b**) impedance signal value produced by non-ferromagnetic metal particle.

By MATLAB calculation, we can get the curve of the real part impedance signal amplitude value of the entire circuit as shown in Figure 5 with the excitation frequency *f* changes.

From the curve in Figure 5, we can see that as the excitation frequency increases, for ferromagnetic particles, the resulting change in the real part of the impedance generated after passing through the detection area increases; for non-ferromagnetic metal particles, the resulting change in the real part of the impedance generated after passing through the detection area decreases. From the foregoing description, it can be seen that the real part of the impedance of the entire circuit increases as the ferromagnetic particles pass through the detection area, and the real part of the impedance of the entire circuit decreases as the non-ferromagnetic particles pass through the detection area. Thence, for the ferromagnetic particles and the non-ferromagnetic particles, the absolute value of the real part of the impedance change produced by the detection area increases with the increase of the excitation frequency.

## 4. Experiments and Discussion

**Figure 6:**
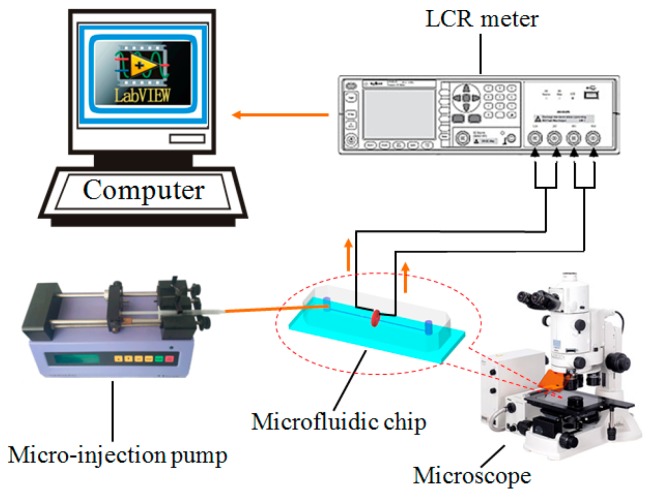
Schematic diagram of impedance detection system.

The impedance detection system is shown in Figure 6. It consists of a micro-injection pump (Harvard Apparatus B-85259, Harvard Apparatus, Holliston, MA, USA), a microscope (Nikon AZ100, Nikon, Tokyo, Japan), a micro fluidic chip, an inductance (L), capacitance (C), and resistance (R) meter (Agilent E4980A, Agilent Technologies Inc., Bayan Lepas, Malaysia) and a computer with LabVIEW software (LabVIEW 2011, National Instruments, Austin, TX, USA).

### 4.1. Experiments Preparations

In the experiment to investigate the effect of excitation frequency on detection, we used iron particles with sizes of 70–80 μm for ferromagnetic particles, and we used copper particles with sizes of 130–140 μm for non-ferromagnetic particles (Hefei Shatai Mechanical and Electrical Technology Co., Ltd., Hefei, Anhui, China). We weighed 4 mg for each particle using a precision balance (Precisa XS255A, Precisa Gravimetrics AG, Luzern, Switzerland), and mixed the particles with 100 mL of Marine hydraulic oil (marine hydraulic oil (The Great Wall L-HM 46, Sinopec Lubricant Co., Ltd., Beijing, China) by oscillator (IKA S25, IKA, Staufen, Germany), then put them into plastic test tube as experiment material.

In the experiment of exploring the lowest limit of chip detection, we used iron particles with size of 20–30 μm, 30–40 μm, 40–50 μm for ferromagnetic particles, and we used copper particles with size of 70–80 μm, 80–90 μm, 90–100 μm for non-ferromagnetic particles.

**Figure 7:**
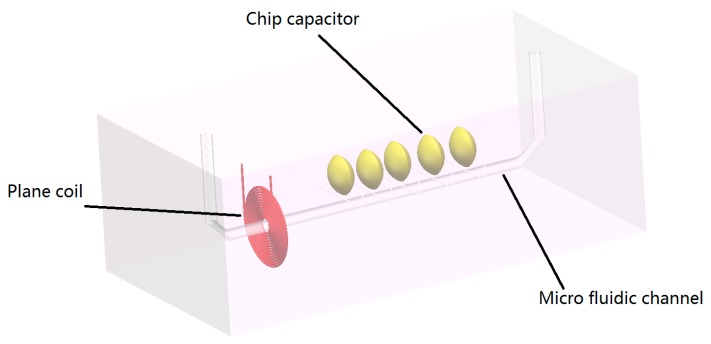
Micro fluidic resonant oil detection chip model figure in the experiments.

In the experiments, in order to ensure that unrelated variables are consistent when detecting particles, we designed the detection chip as shown in Figure 7. This chip contains 1 inductor and 5 capacitors of different sizes. When the excitation frequency is changed, the coil is connected with different capacitor for detection, and the excitation frequency is keeping unchanged. In this way, the particles can be detected in a chip having the same flow channel and the same inductance coil and the external factors interferes can be relatively reduced.

### 4.2. The Influence of Excitation Frequency on Detection

In the experiment, we set the excitation voltage of the chip to 2 V by LCR meter and the injection plastic of the microinjection pump to 40 μL/min. Then put 70–80 μm iron particles and 140–150 μm copper particles into the chip and connected micro-injector pump and finally started the experiment.

**Figure 8:**
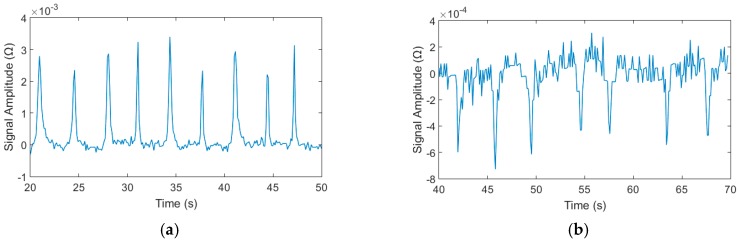
Detection results of iron and copper particles, the excitation voltage was 2 V and the excitation frequency was 0.9 MHz: (**a**) iron particles with sizes ranging from 70–80 μm and (**b**) copper particles with sizes ranging from 140–150 μm.

Among them, the detection results of 70–80 μm iron particles and 140–150 μm copper particles are shown in Figure 8, respectively, at the same voltage and frequency. From Figure 8a,b, we can see that an upward pulse is generated when iron particles pass through the detection area and a downward pulse is generated by copper particles. Thus, we can distinguish the ferromagnetic particles and the non-ferromagnetic particles by the pulse direction judgment.

In the experiments, we used 70–80 μm iron particles and 140–150 μm copper particles to investigate the influence of excitation frequency on detection precision. The size of the chip capacitors and the excitation frequency are shown in Table 1, the excitation frequency is the resonant frequency corresponding to the connected capacitor. And the highest resonant frequency of the chip is 0.9 MHz in laboratory conditions because the minimum capacitor in our laboratory is 0.010 μF.

**Figure 9:**
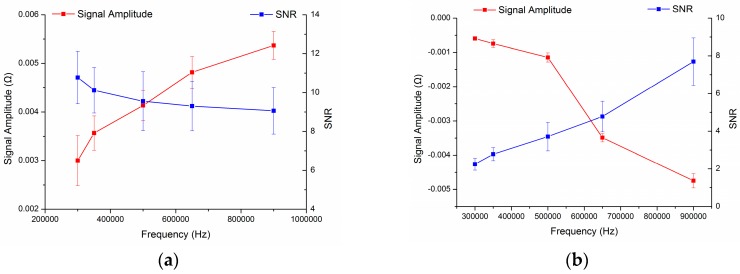
Influence of particle amplitude and signal-to-noise ratio (SNR) with excitation frequency: (**a**) 70–80 μm Iron particles detection results, the blue line was SNR, and the red line was signal amplitude and (**b**) 140–150 μm copper particles detection results, the blue line was SNR, and the red line was signal amplitude.

The experimental results are shown in Figure 9a,b, each average data point and its error bar were evaluated by 10 measurements. From the Figure 9a, we can see that for the same size of iron particles, with the increase of the excitation frequency, the generated pulse size increase, but the signal-to-noise ratio change negatively with the frequency change. From the Figure 9b, we can find that for the same size copper particles, with the increase of the excitation frequency, the generated pulse size increase, and the signal-to-noise ratio (SNR) also change in proportion to the frequency. Furthermore, the amplitude curve of experiments is consistent with theoretical calculations in Figure 5.

### 4.3. Detection Limit of the Detection Chip

From Figure 9 we can also know that the SNR of copper particle detection signal is more effective than the SNR of iron particle detection signal with excitation frequency change. Thus, we have chosen 0.9 MHz as the excitation frequency to investigate the detection lowest limit of the detection chip in the experiments. Moreover, we used iron particles with size of 20–30 μm, 30–40 μm, and 40–50 μm and copper particles with size of 70–80 μm, 80–90 μm, and 90–100 μm as the experiment’s materials.

**Figure 10:**
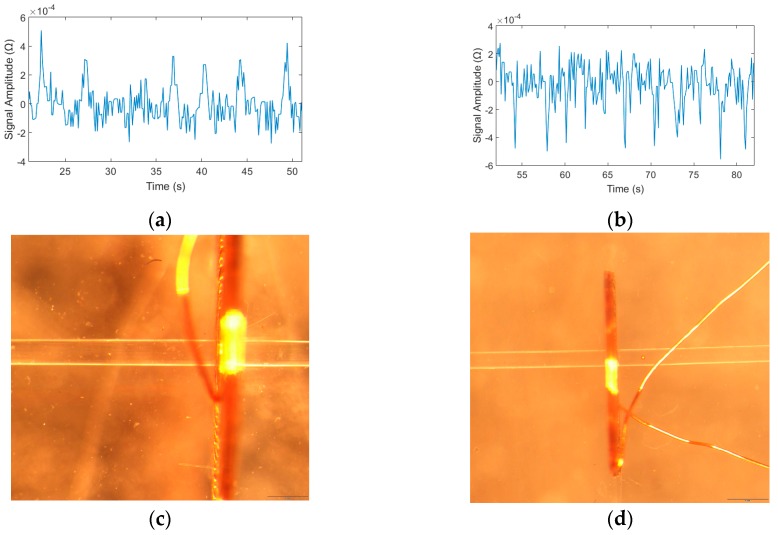
The limitation detection results of iron and copper particles, the excitation voltage was 2 V and the excitation frequency was 0.9 MHz: (**a**) iron particles with sizes ranging from 20–30 μm; (**b**) copper particles with sizes ranging from 70–80 μm; (**c**) 20–30 μm iron particle under microscope; and (**d**) 70–80 μm copper particle under microscope.

Through the experiments, we found that the limitation of detection chip is iron particle 20–30 μm and copper particle 70–80 μm when the excitation voltage is 2 V, the excitation frequency is 0.9 MHz and the injection flow rate is 40 μL/min. The detection signal and the particle figure as shown in Figure 10.

### 4.4. Mixtures Detection of the Detection Chip

**Figure 11:**
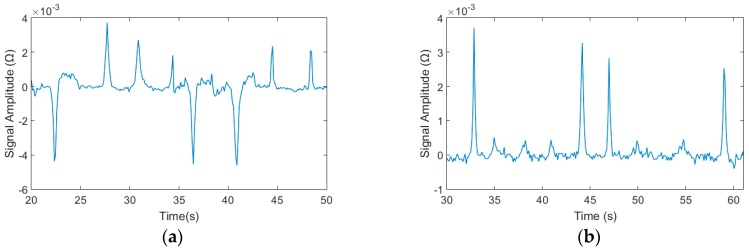
The mixtures detection result of the chip: (**a**) the mixtures of 70–80 μm iron particles and 140–150 μm copper particles results, the positive signals were iron particles, the negative signals were copper particles and (**b**) the mixtures of 20–30 μm and 70–80 μm iron particles detection results, the larger signals were 70–80 μm iron particles, the smaller signals were 20–30 μm iron particles.

The mixtures of 70–80 μm iron particles and 140–150 μm copper particles and the mixtures of 20–30 μm and 70–80 μm iron particles were detected at 0.9 MHz and 2 V condition in experiments to verify that the detection chip can distinguish different size ferromagnetic and non-ferromagnetic particles. And the detection results were shown in Figure 11. The positive signals were generated by iron particles and the negative signals were generated by copper particles in the Figure 10a. The larger positive signals were generated by 70–80 μm iron particles and the smaller were generated by 20–30 μm particles in the Figure 10b.

## 5. Conclusions

A resistance micro fluidic detection chip based on resonant circuit for detecting metal particles in hydraulic oil has been designed in this paper. The experimental results are consistent with the theoretical calculations. The chip would generate a positive resistance pulse when the iron particles pass through the detection area, and the chip would generate a negative resistance pulse when the copper particles pass through the detection area, and the detection signal amplitude increased with resonant frequency. With excitation frequency increased, the signal-to-noise ratio decreased for ferromagnetic particles and increased for non-ferromagnetic particles. Since the highest resonant frequency of the chip is 0.9 MHz in laboratory conditions, we used it as the chip’s excitation frequency to investigate the limitation of detection and detected 20–30 μm iron particles and 70–80 μm copper particles. We can effectively predict the hydraulic system fault by the size of the contaminants and its location to some extent by distinguishing the contaminants. The condition monitoring and fault diagnosis is significance to the ship’s hydraulic system.

The detection accuracy and the throughput of this chip are still limited. In our future works, we will improve the detection accuracy by increase the turns of the coil and improve the throughput by increase the diameter of the fluid channel.

## Figures and Tables

**Table 1:** Different excitation frequencies in the experiments.

Chip Capacitors	Excitation Frequency ^1^
0.10 μF	0.30 MHz
0.068 μF	0.35 MHz
0.047 μF	0.50 MHz
0.022 μF	0.65 MHz
0.010 μF	0.90 MHz

^1^ Excitation frequency *f* = *ω*/*2π*, *ω* is excitation angular frequency.
